# Integrating community engagement with implementation science to advance the measurement of translational science

**DOI:** 10.1017/cts.2022.433

**Published:** 2022-08-01

**Authors:** Christine M. Gunn, Linda S. Sprague Martinez, Tracy A. Battaglia, Rebecca Lobb, Deborah Chassler, Dema Hakim, Mari-Lynn Drainoni

**Affiliations:** 1 Women’s Health Unit, Section of General Internal Medicine, Evans Department of Medicine, Boston Medical Center and Boston University School of Medicine, Boston, MA, USA; 2 Boston University School of Public Health, Department of Health Law, Policy and Management, Boston, MA, USA; 3 The Dartmouth Institute for Health Policy and Clinical Practice, Dartmouth Cancer Center, Lebanon, NH, USA; 4 Macro Department, Boston University School of Social Work, Boston, MA, USA; 5 Boston University Clinical Translational Science Institute, Community Engagement Program, Boston, MA, USA; 6 Section of Infectious Diseases, Department of Medicine, Boston University School of Medicine, Boston, MA, USA; 7 Evans Center for Implementation and Improvement Sciences, Department of Medicine, Boston University School of Medicine, Boston, MA, USA

**Keywords:** Implementation science, National Center for Advancing Translational Sciences (U.S.), community-engaged research, community engagement, program evaluation

## Abstract

This special communication provides an approach for applying implementation science frameworks to a Clinical and Translational Science Institutes (CTSIs) community engagement (CE) program that measures the use of implementation strategies and outcomes that promote the uptake of CE in research. Using an iterative multi-disciplinary group process, we executed a four-phased approach to developing an evaluation plan: 1) creating an **evaluation** model adapted from Proctor’s conceptual model of implementation research; 2) mapping implementation strategies to CTSI CE program interventions that support change in research practice; 3) identifying and operationalizing measures for each strategy; and 4) conducting an evaluation. Phase 2 employed 73 implementation strategies across 9 domains generated by the Expert Recommendations for Implementing Change project. The nine domains were used to classify each CE program implementation strategy. In Phase 3, the group used the Reach, Effectiveness, Adoption, Implementation and Maintenance (RE-AIM) framework to define measures for each individual strategy. Phase 4 demonstrates the application of this framework and measures Year 1 outcomes for the strategy *providing interactive assistance*, which we implemented using a centralized consultation model. This approach can support the CTSA program in operationalizing CE program measurement to demonstrate which activities and strategies may lead to benefits derived by the program, institution, and community.

## Introduction

In the United States, the Clinical and Translational Science Award (CTSA) Program was designed to advance the efficiency of research translation, speeding up the process of bringing discoveries from the laboratory, clinic, and community to policy and practice as a means of improving individual and public health [1]. Community engagement (CE) programs are a required and essential component of all Clinical and Translational Science Institutes (CTSIs), and there are now 50 CTSIs in existence. CE programs seek to extend the capacity of CTSIs to design, implement, and disseminate innovations that benefit communities through bidirectional engagement. This includes partnership development both to identify research priorities and to extend the reach and translation of research findings outside of academia. Indeed, some early evidence suggests CE programs influence an institution’s ability to create and disseminate impactful science when partnering with local health and community organizations [2–7].

**Fig. 1.**
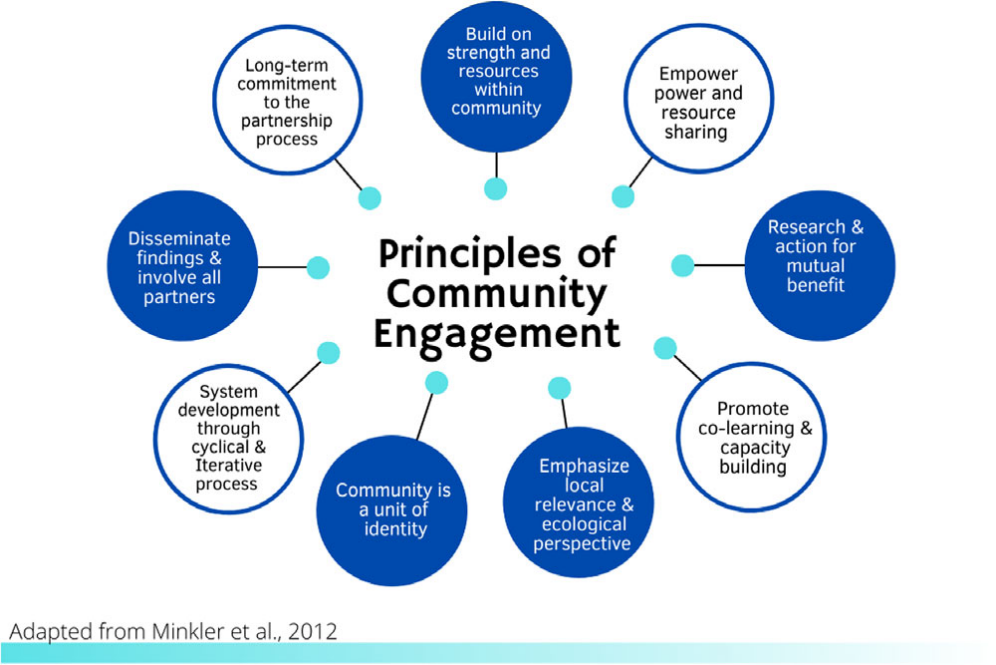
Community engagement principles.

CE itself is an evidence-based practice[8] with established principles to guide public health professionals, health care providers, researchers, and community-based leaders and organizations to authentically engage partners in projects that may affect them (*see* Fig. 1) [9]. However, how CTSA CE programs function to promote successful translation of these principles is not well documented [10], including whether they maintain fidelity to the principles of CE. One study examined how CTSA programs conceptualized and measured CE: It found there were many commonalities in how CE programs defined CE, but measurement was primarily focused on academic products (e.g., number of CE-focused publications, grants applied for or awarded) rather than community-focused outcomes (e.g., partnerships developed) [11]. Despite calls for CTSIs to fill the gaps in understanding about CE science and methods [12], there remain few studies that systematically report on the activities of CE programs and more specifically, the strategies they use to implement CE. These gaps limit widespread dissemination of best practices.

Implementation science (IS) offers a set of frameworks for evaluation that delineate relevant factors in how evidence-based practices, such as CE, are enacted in real-world settings and achieve success [13,14]. At the same time, few CE programs have reported rigorous evaluation frameworks that guide their approach to measurement of CE activities or to examining specific CE program implementation strategies. The contributions of external research team members (community partners**)** are described most often in accounts of prioritizing, developing, and conducting specific research projects and less frequently in reports on dissemination and implementation research [15]. Yet, CE has been framed as a key activity of IS [12]. Based on its relevance to research translation, CTSIs have incorporated some components of IS in recent years, but IS is not commonly integrated into the activities of CTSI programs [16,17]. We therefore sought to use IS frameworks and methods to assess how CE is being implemented in one CTSI.

This study describes the methods used to create and execute a comprehensive evaluation plan for our CTSI CE program rooted in IS. We conducted a four-phase process that iteratively developed methods and metrics for determining success in the implementation of the local CE program. The phases were as follows: 1) create a CE program evaluation framework adapted from Proctor’s conceptual model of implementation research [18]; 2) map implementation strategies to CTSI CE program interventions used to support change in research practice [19]; 3) identify and operationalize measures for each strategy using the Reach, Effectiveness, Adoption, Implementation and Maintenance (RE-AIM) framework [20]; and 4) conduct an evaluation. Below we detail the activities conducted within each phase and report on the final evaluation tools. We also provide an illustrative example of an evaluation of one intervention and associated strategy.

## Materials and Methods

### Background of the CE Program

The specific aims of the Boston University CTSI CE program are to 1) develop new and sustain existing community-academic partnerships; 2) expand CE capacity for researchers and community members and their organizations/systems; and 3) disseminate best practices for CE research approaches. The CE program is comprised of an interdisciplinary team of researchers and practitioners, spanning University Schools across two campuses. Disciplines represented on the team include medicine, epidemiology, social policy, health services research, social work, mental health, and IS.

Core functions of the CE program fall into two main areas: capacity building and partnership development. Capacity-building programs are designed for both internal and external audiences. Programs focus on preparing researchers, trainees, students, community members, and staff to engage in bidirectional partnerships, including raising awareness about the ways in which procedures, policies, and organizational culture can serve as barriers to engagement. Capacity-building activities are designed based on a competency continuum which includes skills that can be deepened over time. Capacity-building activities include guided group networking, case-based learning collaboratives, workshops, educational forums and panels, self-paced modules, the dissemination of educational materials, coaching, and consultation.

Partnership development activities focus on investing in the development of authentic, longitudinal community-academic partnerships that provide a basis for co-creation of research that addresses community priorities. Specific activities include pilot funding for research projects that address a community priority and have community co-leadership; support for development of longitudinal community advisory boards (CABs) that represent diverse stakeholder perspectives to guide research programs; and the implementation of an active practice-based research network in partnership with locally affiliated federally qualified health centers.

### Phase 1: Evaluation Model Development

**Fig. 2.**
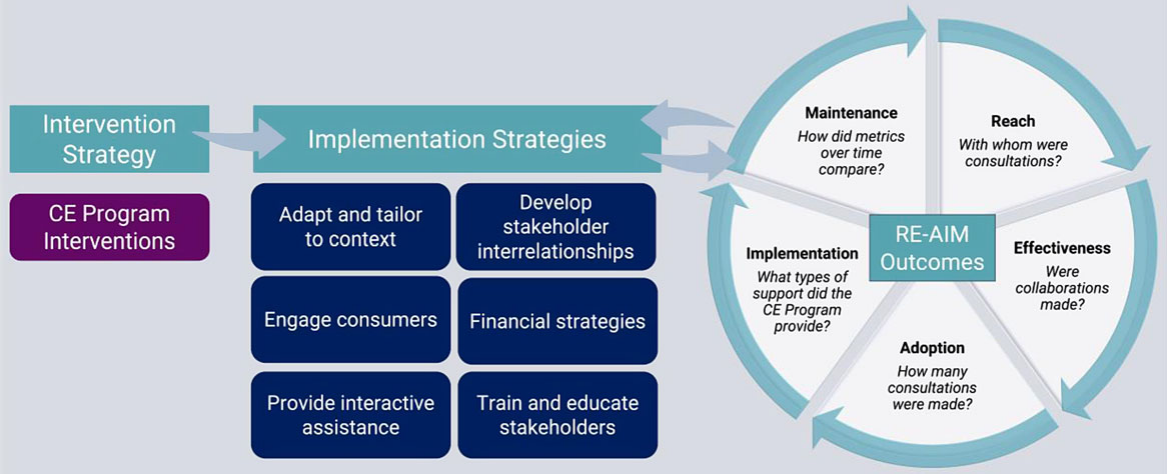
CTSI CE program evaluation framework. CTSI, Clinical and Translational Science Institutes; CE, community engagement.

The process of applying IS frameworks to the CE program began with developing an evaluation model adapted from Proctor’s conceptual model of implementation research [18]. The Proctor model was selected for use as the basis for mapping CE program activities due to its focus on operationalizing the evidence-based practice (in this case, CE as defined by our CE program interventions/activities) and the implementation strategies described below. This phase sought to outline a high-level approach of implementing CE, combined with evaluation to measure program impact. The original Proctor model focuses on outcomes recommended by the Institute of Medicine to measure the impact of evidence-based practices on health outcomes. Since the CE program’s goal was more proximately focused on implementation outcomes, this part of the model was adapted by identifying outcomes that would facilitate transparent reporting and enhance program planning. An evaluation team consisting of an implementation scientist, the two CE program leads who are community-engaged researchers, an expert in evaluation, a health services researcher, a data manager, and program staff was formed. This evaluation team consulted the CE program’s stated aims and the peer reviewed literature to guide an initial evaluation model draft. The draft model was circulated for feedback and discussed. Iterative changes were made over a period of 3–5 months, and the final version represented the group’s consensus on activities and outcomes (Fig. 2). After reaching consensus that an adapted Proctor’s model was appropriate, the next two phases focused on specifying relevant implementation strategies and outcome measures.

### Phase 2: Identification of Implementation Strategies

One of the key mandates of the CE evaluation group was to identify evidence-based implementation strategies to guide the implementation of CE. An implementation strategy is a systematic process to adopt and integrate evidence-based innovations into routine practice [15]. We used the compendium of 73 strategies generated by the Expert Recommendations for Implementing Change (ERIC) project to evaluate methods employed to deliver each CE intervention delivered by our program [21]. The evaluation team assigned each CE program intervention that was described in the evaluation model to at least one of the 73 ERIC implementation strategies to classify which implementation strategy was used to deliver the intervention. For example, the *Communicating to Engage* educational program was classified an example of dynamic education. We then mapped each strategy to one of the Waltz *et al*. clusters of implementation strategies [19]. For *Communicating to Engage*, this was classified under the cluster of “train and educate stakeholders.” Mapping to the Waltz categories brought together related strategies to simplify reporting and allow for coordinated evaluation for strategies used across multiple interventions. Classification was done via an iterative process with program staff and faculty, whereby the rationale for the assigned strategy was presented, reviewed, discussed, and then consensus reached. This process was repeated for each intervention.

### Phase 3: Establishing Evaluation Outcomes

The RE-AIM framework was used to identify the metrics by which the CE program will assess program performance on uptake of CE in research practice at our institution. The RE-AIM framework was chosen to guide this analysis because it has been successfully applied across a range of settings, including public health, clinical, and community contexts [20,22]. The evaluation team, led by the implementation expert, created operational definitions for each element (*Reach, Effectiveness, Adoption, Implementation,* and *Maintenance*). In turn, we defined feasible measures for each RE-AIM element for each implementation strategy. Using “train and educate stakeholders” as an example, *Reach* could be measured as number and types of individuals participating in any of the interventions delivered via this strategy (i.e. monthly seminars, *Communicating to Engage* workshops, *Connecting Communities to Research*, and/or the doctoral-level CE course). Effectiveness and implementation will similarly be measured using a participant-reported evaluation (survey) addressing these RE-AIM domains delivered across all courses. By measuring strategies across the RE-AIM elements, the evaluation will focus on strengths and weaknesses across different phases of implementation that can guide further adaptation of the CE program interventions in an evidence-based manner.

### Data Collection and Analysis Overview

The data collection strategy for program evaluation considered long-term feasibility and sought to minimize burden on program participants, favoring data that could be documented or collected by CE program staff. We also prioritized measures that could be monitored over time to evaluate the *Maintenance* component of RE-AIM (how the intervention strategy RE-AIM metrics compared year-to-year). The evaluation team designed a REDCap form to structure outcome reporting and balance respondent burden with sufficient detail to ensure the collection of meaningful data. With the centralized nature of the consultation model that spanned several strategies (providing interactive assistance, educating and engaging stakeholders, engaging consumers), data could be harmonized and captured in the REDCap database with relative ease. Data from capacity-building activities, including workshops and courses, were self-reported by participants via surveys. Using REDCap surveys, participants who registered for a CE program were asked to provide demographic information (e.g. gender, race, ethnicity) and professional information (e.g. institutional affiliation, role in institution [i.e. trainee, faculty, staff], and experience using community-engaged research methods). Additional data on participation, including minutes of attendance per session, were obtained by CE program staff through Zoom. In addition, the evaluation team records when the CE program connects potential partners and evaluates the effectiveness partnership building via collaboration on papers, grants, or other community-focused products.

Data generated from each intervention strategy for each of the RE-AIM metrics, using both program data and participant surveys, were compiled and summarized using descriptive statistics. Frequency measures (counts, proportions) were used for categorical data. Means, medians, and ranges were reported for continuous measures. On an annual basis, metrics for each intervention strategy continue to be summarized and reviewed by the evaluation team and shared with CTSI leadership. Tables, similar to the one presented below for the “provide interactive assistance,” are used to summarize RE-AIM metrics for each strategy. Annual reporting is used to identify areas where implementation strategies might be enhanced or changed. It also permits the CE program to adapt to increases in capacity over time, such that new interventions or strategies may be introduced. For example, strategies may shift from those that seek to build new partnerships to those that aim to advance ongoing partnerships. The analysis of the maintenance metrics compares Year 1 data to subsequent years and is thus measured only in Years 2–5.

### Phase 4: Application of the Evaluation Framework

To clarify the application of these broad concepts, we describe the methods and evaluation of one strategy: “provide interactive assistance” from the first year of data collection. The CE program operationalized the “provide interactive assistance” strategy as a consultation intervention. The CE program used a centralized consultation model to provide local, tailored technical assistance to teams seeking to incorporate CE into their research. Upon completing any consultation, the program staff entered key information into the database including 1) professional information (e.g., academic affiliation, professional role), 2) reason for the consultation (e.g., advice on a grant proposal, advice on an existing grant-funded project), 3) needs to be fulfilled by the consultation, 4) date the consultation request was made, 5) support and resources provided by the CE program staff (e.g., guidance on developing advisory groups, providing a letter of support, introduction to potential partners, referral to CE Program training activities, etc.), 6) amount of time CE program staff spent working on the consultation, and 7) details of the grant, if applicable (i.e., sponsor, direct costs requested, funding mechanism).

## Results

### Phase 1: Evaluation Model

The evaluation model developed by the evaluation team is detailed in Fig. 2. In short, CE is identified as our intervention strategy. The implementation strategies from Phase 2 are grouped broadly into training and educating stakeholders, engaging consumers, developing stakeholder interrelationships, and providing interactive assistance and financial strategies. Implementation **o**utcomes are organized by RE-AIM constructs with broad definitions for each included in Fig. 2.

### Phase 2: Implementation Strategy Identification

In the second phase, as outlined in the evaluation model, each of the six implementation strategy clusters as defined by Waltz and colleagues was assigned to 11 CE program interventions. Table 1 below outlines each intervention and associated implementation strategies. Using our example intervention of “consultations to seek program guidance,” we see that this intervention grouped to two domains: *train and educate stakeholders* and *provide interactive assistance.* Within these broad domains, the CE program instigated several of the ERIC strategies. They centralized the technical assistance structure, creating unified pathways and forms for initiating consultations. They provided local technical assistance through the consultations, tailoring the length, format, and structure to individual investigators and projects. They also provided ongoing consultation that could last for multiple sessions over the lifetime of a project.

**Table 1.** Boston University CE program activities and associated implementation strategies

CE program interventions	Implementation strategies by cluster^1^
Monthly seminars	Train and educate stakeholders: Make training dynamic Distribute education materials Conduct educational meetings Create a learning collaborative Engage consumers Involve consumers Develop stakeholder interrelationships Capture and share local knowledge Develop academic partnerships
Courses/Workshops: “Communicate to Engage” “Connecting Communities to Research” Doctoral-level course	Train and educate stakeholders: Develop education materials Conduct ongoing training Make training dynamic Distribute education materials Engage consumers Prepare stakeholders to be active participants in CE Research
Distribution of tools through individual communications	Train and educate stakeholders: Distribute education materials
Researcher-initiated consultations to seek CE program guidance	Train and educate stakeholders: Provide ongoing consultation Develop educational materials Provide interactive assistance: Centralized technical assistance structure Provide local technical assistance
Capturing community perspectives	Adapt and tailor to context: Tailor strategies Use data experts Develop stakeholder interrelationships Capture and share local knowledge Use advisory boards and workgroups Train and educate stakeholders Develop educational materials
Facilitate advisory groups	Develop stakeholder interrelationships: Use advisory boards and workgroups
Developing community partnerships	Develop stakeholder interrelationships: Inform local opinion leaders Conduct local consensus discussions Capture and share local knowledge Visit other sites Promote network weaving (MA CTSI Hubs)
Pilot grants	Financial strategies: Fund and contract for innovation
Grant writing	Financial strategies: Access new funding
Promote institutional change in financial systems	Financial strategies: Make billing easier Add/alter incentive or allowance structures
Internal and external dissemination	Engage consumers: Involve community members and researchers Increase demand for CE services Use mass media Develop stakeholder interrelationships: Use an implementation advisor

CE, community engagement; CTSI, Clinical and Translational Science Institutes.

1 Clusters represent domains in Waltz et al., 2015.

### Phase 3: Establishing Outcome Measures

Again, using the *provide interactive assistance* strategy as an illustrative example, we defined *Reach* as the number, proportion, representatives of researchers, and community members who participated in any consultations with CE program members. To assess the adoption of this strategy, we measured the number of consultations delivered. To assess *Reach* of providing local technical assistance, we measured the proportion of consultations conducted with community and academic partners. In measuring *Effectiveness*, we aimed to identify the influence of providing interactive assistance on building capacity and developing partnerships. To this end, we compiled the number and proportion of grants funded following a CE consultation. For *Implementation*, reason for the consultation, types of support provided, types of partnership connections made, and time spent per consultation were compiled. *Maintenance* monitors changes in the above dimensions and measures over time.

### Phase 4: Application of the Evaluation Framework

Results for *providing interactive assistance* for consultations initiated by researchers and community members, organized by RE-AIM elements, are reported in Table 2. All data reported are derived from the REDCap consultation form. As seen in Table 2, 49 consultations were conducted from July 1, 2020 through June 30, 2021. The provision of local technical support was more commonly conducted with academic stakeholders (88%) relative to community stakeholders (12%). The stated reasons for local technical support were distributed between seeking advice on a project or program with existing funding (i.e., governmental, institutional, private) (47%), seeking advice on a project or program that is not yet funded (43%), or other reasons, such as personal development unrelated to a specific community-engaged research project (6%). CE program staff provided a range of technical assistance, including creating CE plans (37%), making introductions to potential partners (33%), establishing a CAB (20%), letters of support (12%), guidance on developing partnerships (10%), and sharing resources by email (10%). The mean time spent per consultation was 86 minutes, although it ranged from 15 to 255 minutes.

**Table 2.** RE-AIM results for providing interactive assistance through CE consultations

RE-AIM domain	Measure	n	%
***Reach***	Consultations completed	49	*N/A*
	**Types of consultations**	**49**	
	Academic	43	88%
	Community	6	12%
***Effectiveness***	Partnerships made resulting in collaboration	TBD*	
	Grant consults that result in grant funding	TBD*	
***Adoption***	Number of unique consultations delivered	49	*N/A*
***Implementation***	**Reason for consult**	**49**	
	Seeking advice on a project/program with existing funding	23	47%
	Seeking advice on a project/program not yet funded	21	43%
	Invited to speak	2	4%
	Other	3	6%
	**Types of support provided by CE programs**	**49**	
	Guidance on CE plan	18	37%
	Introduction to potential partners	16	33%
	Guidance to establish a CAB	10	20%
	Letter of support	6	12%
	Guidance on partnership development	5	10%
	Shared resources by email	5	10%
	Referral to CE Program trainings	4	8%
	Guidance on communicating to a non-research audience	3	6%
	Guidance on study recruitment	3	6%
	CE Program presented at a meeting or event	3	6%
	Guidance on dissemination	2	4%
	Guidance to work with CHCs	1	2%
	Referral to CTSI pilot program	1	2%
	**Types of partnership connections made through consultation**	TBD*	
	Academic		
	Community		
	Other		
		**Mean**	**Range**
	Time spent per consultation (minutes)	86	15 - 255
***Maintenance***	*Change in metrics in comparison to Year 1 (for each metric), measured in future years.*		

CE, community engagement; CTSI, Clinical and Translational Science Institutes; CAB, community advisory board; CHC, Community Health Center.

* TBD, in the process of measuring, will be reported in future years.

## Discussion

We report here on the development and early evaluation of an infrastructure to integrate IS methods and evaluation into a CTSI CE program. The approach is novel in combining IS frameworks to evaluate a CE program. We used the ERIC project implementation strategies to identify relevant implementation strategies employed in a CE program context. Our evaluation used RE-AIM outcome metrics, which have previously not been described in the context of CTSA programmatic evaluations, to our knowledge.

Using consultation data from the 49 consults to date, representing the strategy of *providing interactive assistance*, and the RE-AIM framework, we found the CE program’s reach to favor academic connections vs. community, with 88% of all consultations coming from academic sources. The need to grow the program’s reach outside of academia to promote and deliver technical support to community organizations is an area for growth. A strength of the *providing interactive assistance* strategy was demonstrated by the broadly distributed types of support provided, including planning for grant submissions using CE approaches and supporting implementation of CE methods on existing projects. In using these structured, systematically collected metrics, implementation can shift to support identified opportunities for growth such as those identified here.

Others have similarly recognized the important role that the CTSA program could have in training and disseminating IS as a field. Studies have documented the barriers to engaging in IS in the medical research community. In one recent study of 1767 health researchers, being able to define IS and having attended an IS training were associated with an approximate 3.5 odds of engaging in IS research [23]. The authors identified that increasing familiarity with IS methods and training and educating the research community are key strategies to increasing engagement in implementation research. CTSIs, individually and collaboratively, have potential for supporting the use of IS across the translational spectrum and educating researchers through their programmatic activities. Aligned with these findings, the National Center for Advancing Translational Science, the body funding the CTSA program, has launched a Dissemination and Implementation Research Core, to facilitate cross-CTSI hub collaboration to advance IS as a critical phase in the translational spectrum.

Using implementation strategies as a model for better understanding evidence-based CTSI activities, their relationships to outcomes, and their potential contributions to generalizing program success may advance the mission of CTSIs in translating research into practice. While some institutions have IS expertise, their integration into the CTSI structure and use in evaluating CE have been variable. Identified barriers include a lack of awareness about the value of IS as a field, and having a paucity of senior mentors to train others in IS methods [17]. Building faculty and program expertise in IS may provide a roadmap for CTSIs, and CE programs specifically, to meet long-term goals using best practices in implementation.

This method is not without its limitations. Data here represent a single-center approach, including aims and interventions that were informed by local history and programmatic expertise. The data presented are preliminary and incomplete. While the team designing this evaluation represented multiple academic disciplines, community representation was lacking. Future planning and evaluation will engage community partners to ensure the outcomes of the CE program reflect community priorities. In particular, we plan to design a series of community-driven metrics that overcome the limitations of focusing on implementation measurement alone. For example, we might consider measuring the extent to which study designs or procedures change as a result of engaging community members or track the influence of CE at various phases of research (conception, design, implementation, dissemination). Complementary measures to assess partnerships, such as social network analysis, are also under consideration as a means of quantifying connections made through the CE program.

Our goal was to provide an overview of our approach to integrating frameworks to generate meaningful program evaluation data, rather than present an evaluation of the program itself. Our institution’s robust Evans Center for Implementation and Improvement Sciences, which benefits from institutional resources and support, made this approach to developing an evaluation team with IS expertise viable. This limits generalizability of the approach beyond institutions that do not have local experts on site and/or access to IS expertise. Centralizing expertise and guidance to promote the use of IS in the CTSA program may be a short-term solution to addressing gaps in institutional expertise. Other models include collaborative regional affiliations between CSTIs to support implementation research by sharing expertise [24].

Individual CTSIs should identify the myriad ways in which IS can inform translational science and be used to evaluate CTSI evidence-based activities. The expansion of the use of implementation strategies to implement evidence-based educational, dissemination, and research programs holds great potential for increasing the impact of CSTI infrastructure. To be successful, implementation strategies require tailoring to the institutional context, including aims, activities, and partnerships [25]. The approach outlined here provides a methodology for replication by other translational science programs to systematically document the impact of CE on translational outcomes.
